# Recessive *COL17A1* Mutations and a Dominant *LAMB3* Mutation Cause Hypoplastic Amelogenesis Imperfecta

**DOI:** 10.3390/jpm13101494

**Published:** 2023-10-14

**Authors:** Youn Jung Kim, Yejin Lee, Wonseon Chae, Jung-Wook Kim

**Affiliations:** 1 Department of Pediatric Dentistry & DRI, School of Dentistry, Seoul National University, Seoul 03080, Republic of Korea; ykim71@snu.ac.kr (Y.J.K.); thakdd0119@snu.ac.kr (Y.L.); myurins@snu.ac.kr (W.C.); 2 Department of Molecular Genetics & DRI, School of Dentistry, Seoul National University, Seoul 03080, Republic of Korea

**Keywords:** hereditary, mutation, *COL17A1*, LAMB3, enamel defects

## Abstract

Hereditary conditions that affect tooth enamel in quantity and/or quality are called amelogenesis imperfecta (AI). AI can occur as an isolated condition or as a symptom of a syndrome. An OMIM search with the term “AI” yielded 79 result entries. Mutations in the same gene cause syndromic or non-syndromic AI, depending on the nature of the mutations. In this study, we recruited two AI families and performed mutational analysis using whole-exome sequencing. The proband of family 1, with hypoplastic pitted AI and mild localized atopic dermatitis, had compound heterozygous *COL17A1* mutations (paternal NM_000494.4: c.3598G>T, p.Asp1200Tyr and maternal c.1700G>A, p.Gly567Glu). The proband of family 2, with hypoplastic pitted AI and Jervell and Lange-Nielsen syndrome, had a recurrent *LAMB3* mutation (NM_000228.3: c.3463_3475del, p.(Glu1155Thrfs*51)) in addition to compound heterozygous mutations in the KCNQ1 gene.

## 1. Introduction

Tooth enamel is the most highly mineralized tissue in the human body. Mature enamel has a unique microstructure, with compactly packed hydroxyapatite crystallites [1]. To achieve such hard mechanical properties, highly coordinated biological processes must be well-regulated and function without errors. The assault from certain environmental and/or genetic factors can adversely affect this delicate process, resulting in pathologic findings such as enamel defects [2]. 

Amelogenesis imperfecta (AI) is a collection of rare genetic conditions that affect the tooth enamel quantitatively and/or qualitatively [3]. The original definition of AI is strict and indicates only non-syndromic cases, but syndromic phenotypes have also been identified in some seemingly isolated AI cases [4,5]. 

Clinically, AI can be divided into hypoplastic, hypocalcified and hypomaturation types. Otherwise, it is divided into simply hypoplastic and hypomineralized types [6]. Among these, hypoplastic AI is believed to be caused mainly by defects in secretory-stage ameloblasts. These issues can occur with or without concurrent defects in the hemidesmosome related to skin diseases such as epidermolysis bullosa [7]. 

In this study, we recruited hypoplastic AI families with similar characteristics and performed mutational analysis via whole-exome sequencing. The analysis revealed different genetic etiologies causing AI in the families: compound heterozygous mutations in the *COL17A1* gene and an autosomal dominant truncation mutation in the *LAMB3* gene.

## 2. Materials and Methods

### 2.1. Human Subject Enrollment

The families were recruited with the approval (CRI05003G; 9 December 2021) of the institutional review board of Seoul National University Dental Hospital (SNUDH). The nature of the study was explained to the participating individuals and appropriate informed consent was obtained. Pedigrees were drawn according to the family histories and oral examinations were performed by the communicating author. 

### 2.2. Whole-Exome Sequencing

Genomic DNA was isolated from saliva or peripheral blood samples using a conventional method with the NucleoSpin Blood L kit (Macherey-Nagel GmbH & Co., Düren, Germany) according to the manufacturer’s instructions. The quality and quantity of the isolated genomic DNA were measured. Probands of the families were subjected to whole-exome sequencing. Exome capturing and the generation of paired sequencing reads were performed at Macrogen (Seoul, Republic of Korea), Yale Center for Mendelian Genomics (West Haven, CT, USA), and Theragen Etex Bio Institute (Suwon-si, Gyeonggi-do, Republic of Korea). 

### 2.3. Bioinformatic Analysis 

Obtained sequencing reads were aligned to the reference sequences and analyzed in order to obtain a list of variants [8]. Adapter sequences from the paired sequencing reads were removed; then, paired-end sequencing reads were aligned to the reference human genome assembly hg38 and a series of bioinformatic tools were used to obtain a list of nucleotide changes, including small deletions/insertions. The list was annotated against the dbSNP build 147 database and annotated variants were filtered using the criteria for a minor allele frequency of 0.01.

### 2.4. Sanger Sequencing

Identified mutations were confirmed via Sanger sequencing at the DNA sequencing center at Macrogen. The primer pairs for the *COL17A1* c.3598G>T variant were 5’-CTTTCTTGGACCCCACTTCT-3’ (forward) and 5’-GCACTGTACAGGCTCCAGG-3’ (reverse). The primer pairs for the *COL17A1* c.1700G>A variant were 5’-TTCTTCCCATACCATGATCC-3’ (forward) and 5’-ACACCTGTCCCATCTGTTGT-3’ (reverse). The primer pairs for the *LAMB3* c.3463_3475del variant were 5’-CTGGAGAGGCATGAAGCTG-3’ (forward) and 5’- GCTGCAGCTCAGGGTAATCT-3’ (reverse).

## 3. Results

### 3.1. Family 1

**Figure 1:**
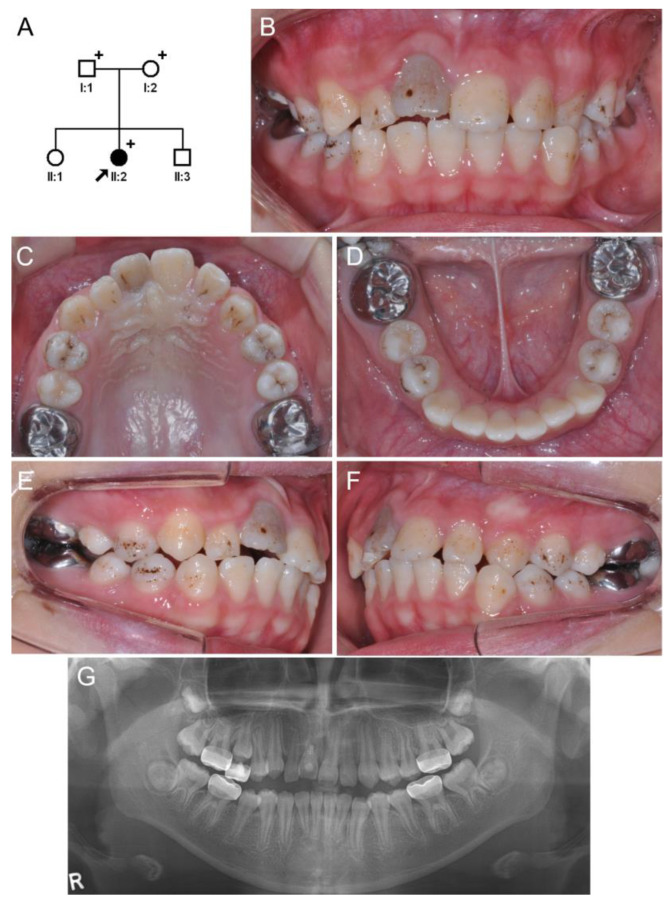
Pedigree, clinical photos, and panoramic radiograph of family 1. (**A**) Pedigree of family 1. The black symbol indicates the affected individual, and the proband is indicated by a black arrow. Plus signs above the symbols indicate participating individuals. (**B**–**F**) Clinical photos of the proband at age 12 years. The maxillary right central incisor is discolored gray and ankylosed due to traumatic avulsion. Almost all teeth surfaces have irregular pits with external staining. (**G**) Panoramic radiograph of the proband at age 12 years. The enamel thickness seems within the normal range in general. There are no other abnormalities in the dentin or root forms.

A girl aged 9 years and 7 months visited the dental emergency room for the treatment of an avulsed maxillary right central incisor due to an injury received while falling down. After the initial treatment, including the re-insertion and fixation of the avulsed tooth, she was referred to the department of pediatric dentistry at SNUDH. Oral examination revealed irregular hypoplastic pits on the enamel surface in almost all teeth. She was a second daughter from a non-consanguineous marriage (Figure 1), and the other family members do not have enamel defects. She had mild, but not severe, atopy-related skin regions around the knees and elbows. Additionally, she reported that her mother also had similar atopic lesions. Otherwise, family members had no other remarkable medical history and conditions.

**Figure 2:**
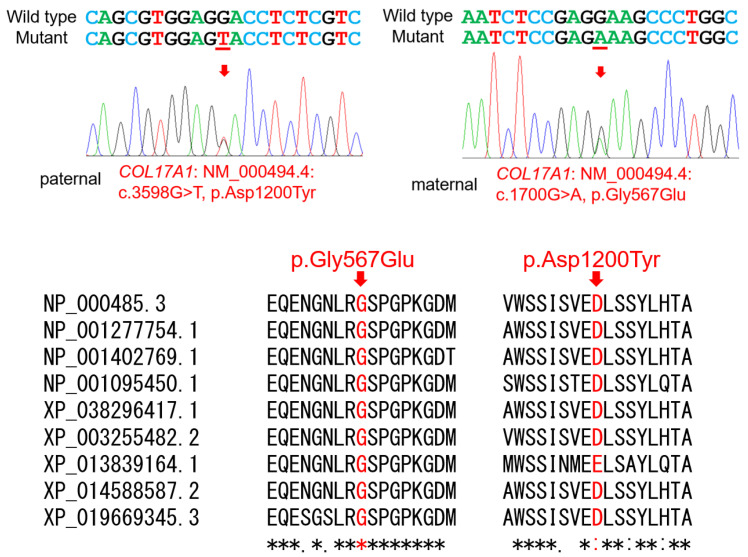
Sequencing chromatograms. Normal nucleotides (with a black bar above the sequence) in the upper chromatogram of the normal individual, IV:2, are deleted, and the novel nucleotides (with a red bar above the sequence) are inserted into the lower chromatogram of the proband, IV:4. The deletion and insertion mutations are described as c.2680_2699delinsACTATAGTT (NM_182758.4) and p.(Ser894Thrfs*15).

Mutational analysis revealed compound heterozygous missense variants with uncertain significance in the *COL17A1* gene. This played the role of encoding the alpha chain of type 17 collagen (Table 1) (Figure 2). This gene has been known to be associated with intermediate junctional epidermolysis bullosa-4 (JEB4, OMIM #619787) in autosomal recessive inheritance but is also associated with epithelial recurrent erosion dystrophy (OMIM #122400) in autosomal dominant inheritance [9,10]. The proband inherited a transversion mutation from the father, which changed a highly conserved aspartic acid into tyrosine at codon position 1200 (NM_000494.4: c.3598G>T, p.(Asp1200Tyr)). The proband additionally acquired a transition mutation from the mother, which changed a completely conserved glycine to glutamic acid at codon position 567 (c.1700G>A, p.(Gly567Glu)). The paternal mutation was novel and not listed in the dbSNP (https://www.ncbi.nlm.nih.gov/snp/, accessed on 1 July 2023) and GnomAD (https://gnomad.broadinstitute.org/, accessed on 1 July 2023) databases. The CADD score was 27.7, and in silico predictions performed using PolyPhen2 and SIFT, respectively, were probably damaging (score: 0.998) and deleterious (score: 0.00). The maternal mutation was also novel. The CADD score was 28.6, and in silico predictions were made. When measured using PolyPhen2, it was considered damaging (score: 1). Assessed using SIFT, it was found to be deleterious (score: 0.00).

### 3.2. Family 2

**Figure 3:**
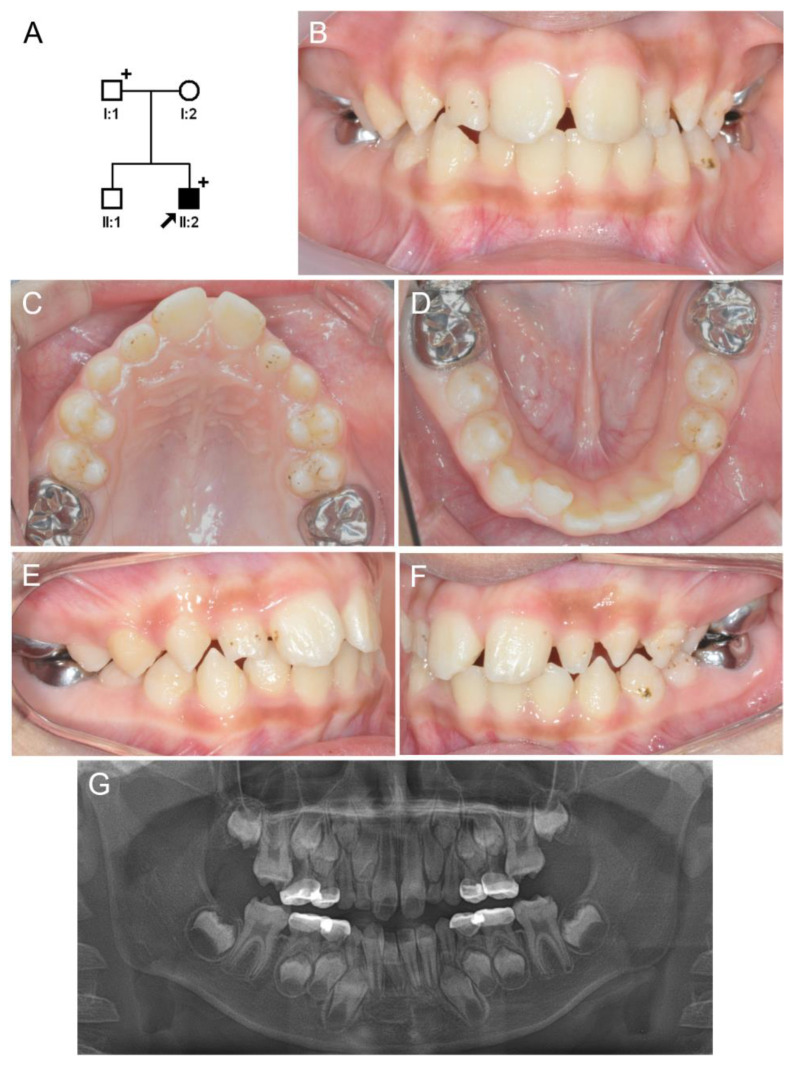
Pedigree, clinical photo, and panoramic radiograph of family 2. (**A**) Pedigree of family 2. The black symbol indicates the affected individual, and the proband is indicated by a black arrow. A plus sign above the symbol indicates participating individuals. (**B**–**F**) Clinical photo of the proband at age 11 years. His mild hypoplastic enamel has irregular hypoplastic pits with staining. (**G**) A panoramic radiograph of the proband at age 7 years shows an ectopic eruption of the bilateral maxillary first molars. The enamel looks hypoplastic, especially in the first molars.

The proband of family 2 was for a boy aged 7 years and 9 months who had been referred for the treatment of a bilateral ectopic eruption of the maxillary first permanent molars (Figure 3). His mild hypoplastic enamel surface had irregular pits and grooves. He suffered from long QT syndrome and congenital sensorineural deafness and had been diagnosed with Jervell and Lange-Nielsen syndrome a year prior. Candidate gene sequencing confirmed the syndrome by identifying compound heterozygous mutations in the KCNQ1 gene [11]. 

**Figure 4:**
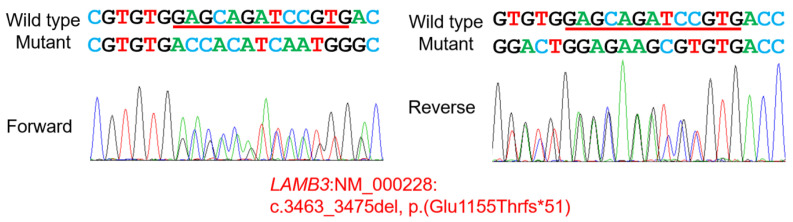
Sequencing chromatograms. Heterozygous deletion of GAGCAGATCCGTG (with a red bar underneath the sequence) in the proband is shown on the left (forward) and right (reverse) sequencing chromatograms.

**Figure 5:**
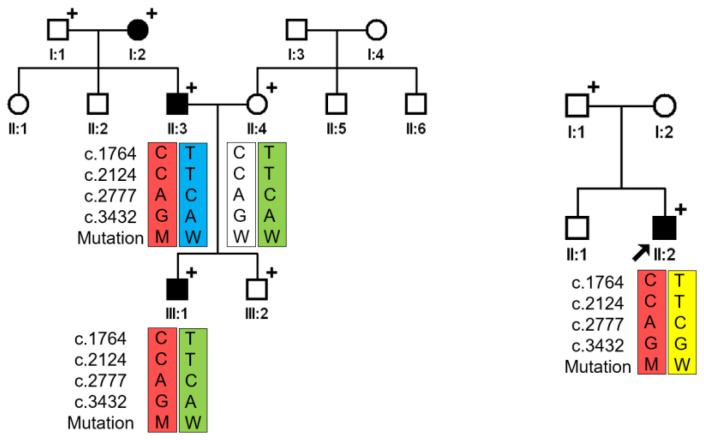
Haplotypes of previously reported family and the family 2 in this study. Disease allele (red color) seems to be identical by descent. Paternal alleles are shown on the left and maternal alleles (green) are shown on the right. Locations of the variants on the list are based on mRNA sequence NM_000228.3.

Mutational analysis for the enamel defect revealed a recurrent frameshift pathogenic variant in the *LAMB3* gene. This gene has been associated with intermediate and severe junctional epidermolysis bullosa (JEB1A and JEB1B, OMIM #226650 and #226700) in autosomal recessive inheritance [12]. Later, it was identified that some specific heterozygous mutations cause AI, Type IA (autosomal dominant hypoplastic AI, OMIM #104530) [13,14]. The identified mutation was a deletion that caused a frameshift in the last exon (NM_000228.3: c.3463_3475del, p.(Glu1155Thrfs*51)) (Figure 4), thereby escaping from the nonsense-mediated mRNA decay system (NMDS). This mutation was previously identified in a Korean family and exhibited variable expressivity [15]. Therefore, even though this family is seemingly unrelated to the original family, this mutation could have originated from a common ancestor. Haplotype analysis showed the same disease allele within the limitation of the study (Figure 5).

## 4. Discussion

The proband of family 1 had irregular pitted hypoplastic AI and localized atopic dermatitis, and compound heterozygous missense *COL17A1* mutations were identified. Even though the compound heterozygous missense mutations suggest skin fragility in the JEB, the relationship to the localized atopic dermatitis must be further characterized. Enamel defects, hypoplastic pitting, and sometimes discolored teeth are among the syndromic phenotypes related to the JEB [16]. Therefore, it is plausible that the mutations identified in family 1 could be hypomorphic mutations that cause the hypoplastic pitted AI phenotype without JEB development.

The proband of family 2 had two separate genetic conditions at once: Jervell and Lange-Nielsen syndrome caused by compound heterozygous mutations in the *KCNQ1* gene and hypoplastic pitted AI caused by an NMDS-escaping truncated heterozygous *LAMB3* mutation [15]. Next-generation sequencing made it possible to distinguish the two separate genotype–phenotype relationships. An incorrect phenotype could be misclassified through traditional genetic analyses as part of an unrelated syndrome [6].

The introduction of next-generation sequencing has led to remarkable advances in modern genetics. Understanding the comprehensive genetic information of individuals or populations provides a fundamental background for personalized medicine. Even with targeted panel sequencing, numerous variants are annotated. Therefore, the correct interpretation of the actual pathologic variant(s) is crucial for understanding normal and pathologic conditions, especially in genetic counseling for marriage and pregnancy [17].

Nonsense or frameshift mutations, resulting in an early translation stop codon, produce short or truncated toxic protein with dominant-negative or gain-of-function effects if the NMDS does not degrade the mutant mRNA transcript [18]. A premature stop codon (PTC) in the early exon is detected and destined for degradation after the pioneer round of translation [19]. However, if the PTC is located in the last exon or within the 50–55 nucleotides from the last exon–exon junction (from the 3’ end of the penultimate exon), the NMDS cannot distinguish the PTC-harboring mRNA transcript; therefore, the truncated protein is generated [20]. 

In autosomal dominant diseases, heterozygous mutations can cause pathologic phenotypes due to haploinsufficiency (null mutation by complete NMD or leaky mutation by incomplete NMD), lack of proper functional activity, dominant-negative effect, etc. In most recessive diseases, however, a loss of function (LOF) mutation in a single allele does not induce clinical pathological conditions, even if the mutation is a null mutation [21]. Likewise, the carriers do not develop the disease if the mutation is hypomorphic. In the recessive condition, hypomorphic mutations in both alleles sometimes cause a less severe disease compared to a hypomorphic mutation in an allele, LOF mutation in the other allele, or LOF mutations in both alleles [22].

Non-syndromic AI caused by heterozygous *LAMB3* mutations has common features: the mutations are predicted to escape the NMDS, and truncated C-terminus proteins are produced [13,14,15,23,24,25,26,27]. These mutations introduce a dominant-negative effect, causing disease. However, heterozygous mRNA transcripts containing a nonsense or frameshift mutation in the early exons, except for the last two, are degraded by the NMDS [19,20]; therefore, a dominant-negative effect is often not generated. Otherwise, evidence to prove its dominant-negative effect should be provided if scientists are to suggest it as a pathological variant in an autosomal dominant condition.

## 5. Conclusions

In this study, we recruited two localized hypoplastic AI families and performed mutational analysis. The analysis revealed that the proband of family 1 had autosomal recessive hypoplastic AI and mild localized atopic dermatitis caused by hypomorphic *COL17A1* missense mutations. We found that the proband of family 2 had Jervell and Lange-Nielsen syndrome, caused by compound heterozygous mutations in the *KCNQ1* gene, as well as hypoplastic pitted AI caused by an NMDS-escaping truncated heterozygous *LAMB3* mutation.

## Figures and Tables

**Table 1:** Statistics for exome sequencing.

Sample	Total Reads	Mapping Rate (%)	Median Target Coverage	Coverage of Target Region (%)	Fraction of Target Covered with at Least	
					20X	10X
Family 1-II:2	108,198,568	99.8	70	92.3	83.6	88.1
Family 2-II:2	103,344,371	99.9	101	99.4	97.2	98.8

## Data Availability

The data presented in this study are openly available in ClinVar (http://www.ncbi.nlm.nih.gov/clinvar (accessed on 5 September 2023)), Accession ID: SCV004031447, SCV004031446, and SCV004031444.
